# Congenital Lumbar Hernia: A 15-Year Experience at a Single Tertiary Centre

**DOI:** 10.1155/2016/7162475

**Published:** 2016-11-22

**Authors:** K. N. Rattan, Arushi Agarwal, Ankur Dhiman, Ananta Rattan

**Affiliations:** Department of Pediatric Surgery, PGIMS Rohtak, Haryana, India

## Abstract

*Aim*. Congenital lumbar hernia is an uncommon anomaly with only few cases reported in the English literature. This study was done to study the incidence, age at presentation, sex, associated anomalies, surgical management, and postoperative morbidity and mortality of congenital lumbar hernia in pediatric patients.* Methods*. Retrospective analysis of all patients of CLH over a period of 15 years (January 2000 to December 2015) was analyzed.* Results*. A total of 14 patients were encountered in this series. All presented within first 2 years of age. 12 were males and 2 were females. All of them presented with swelling in lumbar region. 13 were unilateral and 1 was bilateral. Left sided hernia was observed in 2 cases only. Lumbocostovertebral syndrome was found in all the patients in addition to other rare anomalies. All cases were managed with open surgical repair. Wound infection was seen in 2 cases. There was no mortality in our series.* Conclusion*. CLH is very rare among hernias. Surgery should be carried out within 1 year of age. For a defect of <5 cm, primary repair is done. For a defect of >5 cm, meshplasty should be considered. Prognosis is excellent.

## 1. Introduction

Approximately 20% of lumbar hernias are congenital in nature, while remainder are acquired [1]. Congenital lumbar hernia (CLH) is associated with a multitude of congenital anomalies involving various other organ systems of the body. These involve the ribs, spine, muscles, kidneys, and spinal meninges. Less than 50 cases of congenital lumbar hernia associated with other congenital anomalies have been reported in English literature making it a rare entity [1, 2].

## 2. Aim

We aim to study the incidence, age at presentation, sex, associated anomalies, surgical management, and postoperative morbidity and mortality of congenital lumbar hernia in pediatric patients.

## 3. Materials and Methods

Retrospective analysis of all patients admitted with congenital lumbar hernia in the Department of Pediatric Surgery at Pt. B. D. Sharma, PGIMS Rohtak, Haryana, India, over a period of 15 years from January 2000 to December 2015 was analyzed for sex, age, associated anomalies, site of hernia, clinical presentation, radiological investigation, surgical management, and postoperative morbidity and mortality.

## 4. Results

**Figure 1:**
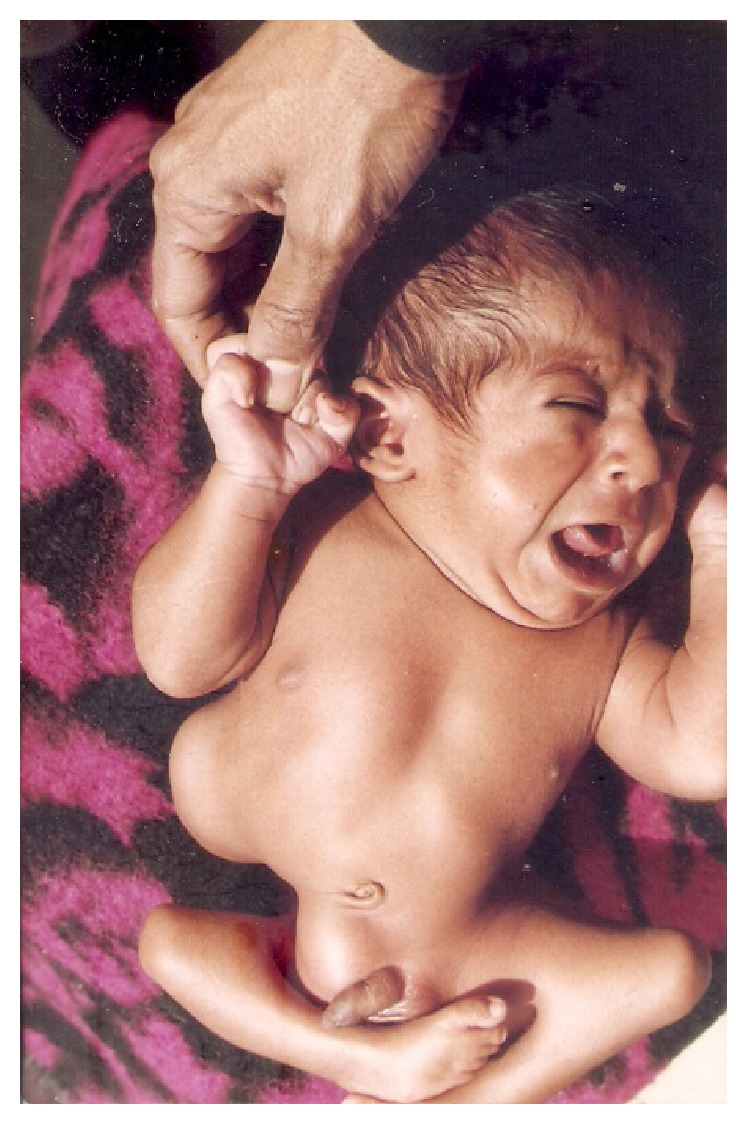
Right congenital lumbar hernia with severe musculoskeletal deformity and right inguinal hernia.

**Figure 2:**
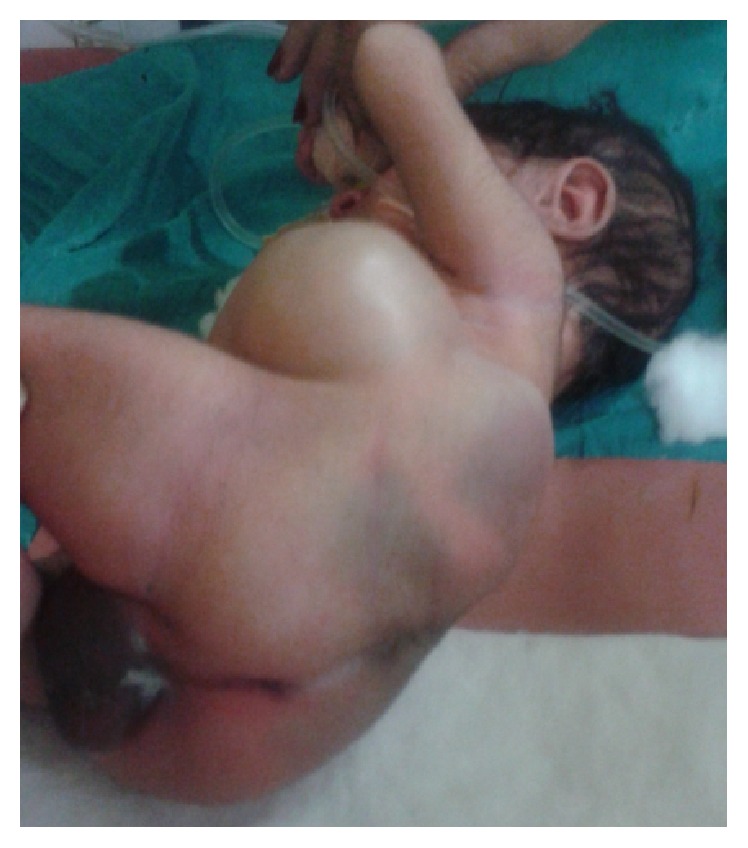
Left congenital lumbar hernia with ARM and vertebral defect.

**Figure 3:**
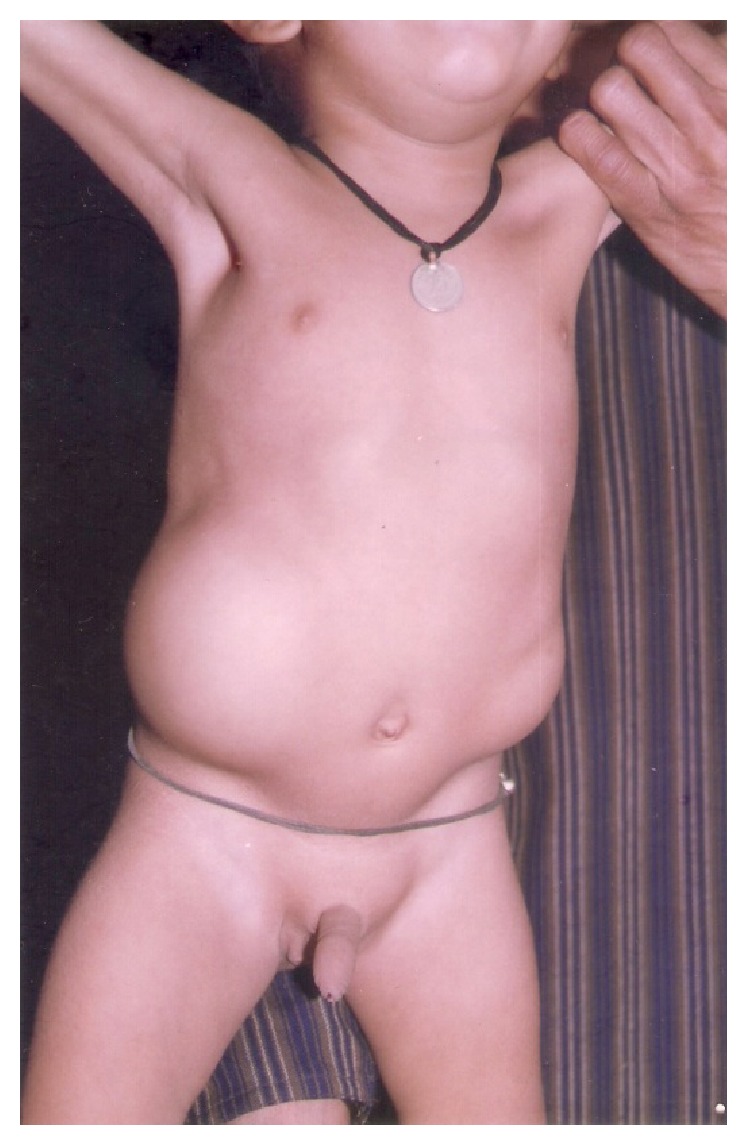
Bilateral congenital lumbar hernia.

**Figure 4:**
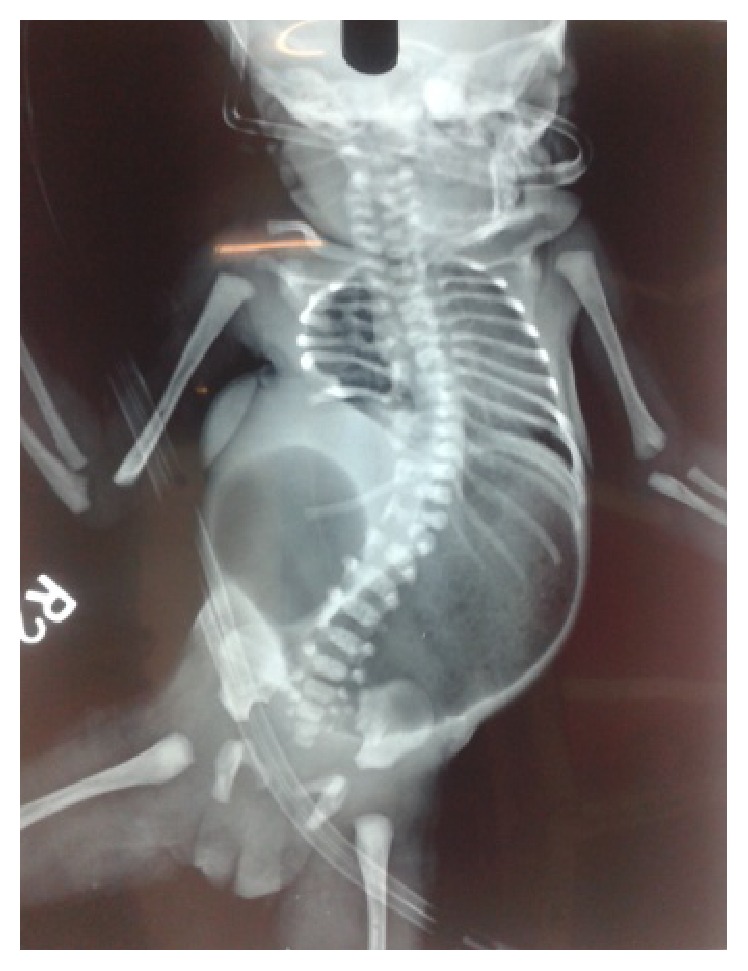
X-ray showing congenital lumbar hernia associated with duodenal atresia and lumbocostovertebral syndrome.

A total of 14 patients were encountered in this series. All presented within first 2 years of age. 12 were males and 2 were females. All the patients were diagnosed clinically with reducible swelling in lumbar region present since birth, which increased in size on crying or coughing (Figure 1). 13 were unilateral and 1 was bilateral (Figure 3). Only 2 of the hernias were found in inferior triangle of Petit. Left sided hernia (Figure 2) was observed in 2 cases only (shown in Table 1). X-ray abdomen including spine showed lumbocostovertebral syndrome (Figure 4). USG abdomen was done to identify the contents of the hernial sac. MRI and CT spine were also done to look for the anomalies of spinal cord. Lumbocostovertebral syndrome was found in all the patients in addition to other rare anomalies (shown in Table 2). Echocardiography was done to rule out associated cardiac anomalies.

**Figure 5:**
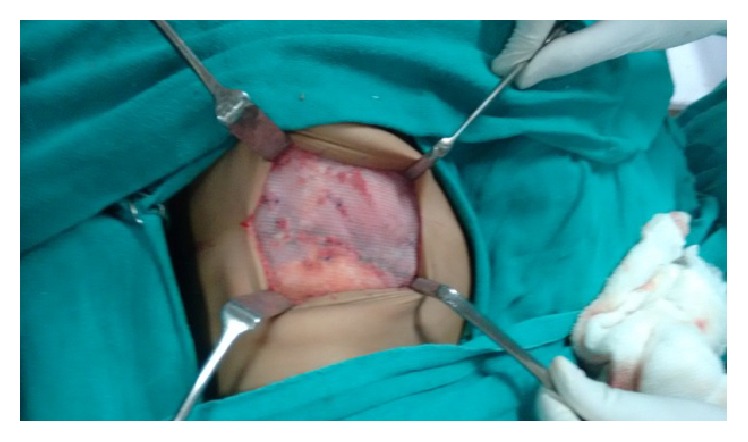
Open surgical repair with meshplasty.

After routine investigations, patients were taken up for surgery. 2 of the patients are waiting for the surgery as they are less than 6 months of age. 1 patient was lost to follow-up and hence could not be operated on. All the rest of the cases were managed with open surgical repair. Surgery was done under general anaesthesia. Skin incision was given along with the sac and the defect was identified. Sac was opened; contents were identified and reduced. In most of the cases, small intestine and large intestine comprised the hernial sac contents. However in one of the cases kidney was the sac content. Defect was repaired using local healthy fasciomuscular tissue. In our study, 2 cases had large defect (7 × 7 cm), where meshplasty was done with drainage (Figure 5). In postoperative period, patients were started orally after 1 day and discharged after day 7. Stitches were removed on day 10. In case of meshplasty, drain was removed after 72 hours. Wound infection was seen in 2 cases which were managed with local dressing and antibiotics. There was no mortality in our series. All the operated on cases are doing well on follow-up with no evidence of recurrence of hernia. They are being followed up in orthopaedics also for the correction of disability caused by lumbocostovertebral syndrome.

## 5. Discussion

Congenital lumbar hernias usually present as large compressible soft-tissue mass in the lumbar region. The lumbar region is divided into 2 triangles: inferior and superior [3]. Inferior lumbar triangle is composed of the iliac crest inferiorly and the margins of two muscles—latissimus dorsi (posteriorly) and external abdominal oblique (anteriorly). The floor of the inferior lumbar triangle is the internal abdominal oblique muscle. It is named after French surgeon Jean Louis Petit (1674–1750). Superior lumbar triangle is formed medially by the quadratus lumborum muscle, laterally by the internal abdominal oblique muscle, and superiorly by the 12th rib. The floor of the superior lumbar triangle is the transversalis fascia and its roof is the external abdominal oblique muscle. It is named after physician Joseph Grynfeltt (1840–1913). Lumbar hernias are more common in this triangle.

In our study also most of the hernias were in superior triangle, while only 2 were in inferior triangle. The hernial sac may be empty or may contain retroperitoneal fat, the bowel, kidney, omentum, colon, stomach, ovary, spleen, appendix, and kidney. In the present study, most common sac contents were gut and retroperitoneal fat. In 1 case, however, kidney was the content. From the etiopathogenic point of view, lumbar hernias may be congenital—presenting soon after birth or in the first years of life, or acquired (spontaneous, posttraumatic, postoperative, and a postregional suppurative process) [1, 4]. About 20% of the lumbar hernias are congenital and the rest are classified as acquired [1].

It is proposed that a single somatic mutation in early embryogenesis, possibly due to transient anoxia, causes derangement of lumbar muscles and aponeuroses resulting in herniation leading to congenital lumbar hernia; however, the causes of congenital hernias have not been completely defined [5]. The congenital form may be isolated, although it often occurs in association with the lumbocostovertebral syndrome, described by Touloukian [5] in 1972, which includes one or more of the following anomalies: hemivertebra, rib abnormalities (absence, hypoplasia, fusion, and gap), aplasia of dorsolumbar muscles, and scoliosis eventually with the convex curve on the site of hernia. Other associated abnormalities reported include absence of the right kidney, eventration of the right hemidiaphragm, inguinal hernia, malrotation of the gut, and ARM. In our study lumbocostovertebral syndrome was present in all the cases along with other associated anomalies.

A roentgenogram of the lumbar region may detect air filled bowel loops in the sac, whereas CT and ultrasonography are most useful diagnostic tools especially to detect solid organs in the sac [6]. Possible complication of lumbar hernia may include incarceration but it is unusual because of the broad neck of sac. Early elective surgical intervention to close the defect along with repair using local tissues is the modality of choice [1, 7]. The treatment of lumbar hernia is surgical and should be performed in the first year of life [8, 9]. If the size of the defect is large or if there is extensive muscular hypoplasia, prosthetic material may be required for surgical repair [1, 10]. Prognosis is good and recurrence is rare [9].

Mainly all the cases of CLH reported in literature include case reports of single cases and most of them did not have any other associated anomaly. In literature, there are only two major case series on CLH conducted so far, and they have been compared with our study here. A. Wakhlu and A. K. Wakhlu described nine patients with CLH [1]. Unusual features in this study included the relatively high incidence of inferior lumbar hernia, contrary to our study where only 2 of the hernias were found in inferior triangle of Petit. Other unusual findings in their study were presentation at the age of 6 years in one case, and an association with hydrometrocolpos and anorectal malformation. In seven patients the hernia could be repaired successfully. Sharma et al. described 18 patients with CLH [11]. There were two patients with bilateral hernias in this series, similar to our study, where 1 patient had bilateral hernia. All cases studied by Sharma et al. were in the age group of 1 day to 6 years. All the patients were operated on by open technique in this series. Primary repair was done in 14 patients and prosthetic meshplasty in two. Two patients could not be operated on. There was no evidence of recurrence. In our study, we have described 14 patients with CLH out of whom 11 have already been successfully operated on by open technique, without any recurrence, while surgery is awaited in 2 and 1 patient was lost to follow-up.

## 6. Conclusion

After this study of 14 patients, we concluded that congenital lumbar hernia is very rare among hernias. It mostly affects males. Right sided hernia is much more common, while bilateral hernia is very rare. Most hernias occur in inferior triangle. Lumbocostovertebral syndrome is the most common associated anomaly. They must be diagnosed and managed early to prevent obstruction and strangulation of contents. Surgery should be carried out within 1 year of age. For a defect of <5 cm, primary repair is done. For a defect of >5 cm, meshplasty should be considered. Long term follow-up is required. Prognosis is excellent. Orthopaedic follow-up is also important in case of lumbocostovertebral syndrome.

## Figures and Tables

**Table 1:** Site of lumbar hernia.

Site of hernia	Number of cases
Unilateral (U/L)	13
(i) Left sided	2
(ii) Right sided	11
Bilateral (B/L)	1

**Table 2:** Associated anomalies.

S. number	Associated anomalies	Number of cases
1	Lumbocostovertebral syndrome	14
2	Anorectal malformation (ARM)	2
3	Right inguinal hernia	1
4	Congenital talipes equinovarus (CTEV)	1
5	Multiple musculoskeletal defects	1
6	Duodenal atresia	1
7	Congenital heart disease	1
8	Solitary kidney	1

## References

[1] Wakhlu A., Wakhlu A. K. (2000). Congenital lumbar hernia. *Pediatric Surgery International*.

[2] Pelaez Mata D. J., Alvarez Munoz V., Fernandez Jimenez I., Garcia Crespo J. M., Teixidor de Otto J. L. (1998). Congenital lumbar hernia. *Cirugia Pediatrica*.

[3] Guillem P., Czarnecki E., Duval G., Bounoua F., Fontaine C. (2002). Lumbar hernia: anatomical route assessed by computed tomography. *Surgical and Radiologic Anatomy*.

[4] Coniglio G., Coniglio l., Coniglio R. (1965). Ernie lombari congenite e acquisite. Contributo clinico: 3 casi. *Policlinico*.

[5] Touloukian R. J. (1972). The lumbocostovertebral syndrome: a single somatic defect. *Surgery*.

[6] Boker M. E., Wenerth J. L., Andiani R. T. (1987). Lumbar hernia: diagnosis by CT. *American Journal of Roentgenology*.

[7] Stamatiou D., Skandalakis J. E., Skandalakis L. J., Mirilas P. (2009). Lumbar hernia: surgical anatomy, embryology, and technique of repair. *The American Surgeon*.

[8] Fakhry S. M., Azizkhan R. G. (1991). Observations and current operative management of congenital lumbar hernias during infancy. *Surgery Gynecology and Obstetrics*.

[9] Hancock B. J., Wiseman N. E. (1988). Incarcerated congenital lumbar hernia associated with the lumbocostovertebral syndrome. *Journal of Pediatric Surgery*.

[10] Akçora B., Temiz A., Babayiğit C. (2008). A different type of congenital lumbar hernia associated with the lumbocostovertebral syndrome. *Journal of Pediatric Surgery*.

[11] Sharma A., Pandey A., Rawat J., Ahmed I., Wakhlu A., Kureel S. N. (2012). Congenital lumbar hernia: 20 years' single centre experience. *Journal of Paediatrics and Child Health*.

